# Pro-Inflammatory Cytokines: Potential Links between the Endocannabinoid System and the Kynurenine Pathway in Depression

**DOI:** 10.3390/ijms22115903

**Published:** 2021-05-31

**Authors:** Ferenc Zádor, Sâmia Joca, Gábor Nagy-Grócz, Szabolcs Dvorácskó, Edina Szűcs, Csaba Tömböly, Sándor Benyhe, László Vécsei

**Affiliations:** 1 Institute of Biochemistry, Biological Research Center, H-6726 Szeged, Hungary; zador.ferenc@gmail.com (F.Z.); dvoracsko.szabolcs@brc.hu (S.D.); szucsedina7@gmail.com (E.S.); tomboly.csaba@brc.hu (C.T.); benyhe.sandor@brc.hu (S.B.); 2 Department of Biomedicine, Aarhus University, 8000 Aarhus C, Denmark; sjoca@biomed.au.dk; 3 Faculty of Health Sciences and Social Studies, University of Szeged, H-6726 Szeged, Hungary; gabor.balazs.nagy@gmail.com; 4 Albert Szent-Györgyi Clinical Center, Department of Neurology, Faculty of Medicine, University of Szeged, H-6725 Szeged, Hungary; 5 Department of Medical Chemistry, University of Szeged, H-6720 Szeged, Hungary; 6 Doctoral School of Theoretical Medicine, Faculty of Medicine, University of Szeged, H-6720 Szeged, Hungary; 7 MTA-SZTE Neuroscience Research Group, University of Szeged, H-6725 Szeged, Hungary; 8 Department of Neurology, Interdisciplinary Excellence Center, University of Szeged, H-6725 Szeged, Hungary

**Keywords:** depression, endocannabinoid system, kynurenine pathway, pro-inflammatory cytokines, cannabis, synthetic cannabinoids, kynurenines

## Abstract

Substance use/abuse is one of the main causes of depressive symptoms. Cannabis and synthetic cannabinoids in particular gained significant popularity in the past years. There is an increasing amount of clinical data associating such compounds with the inflammatory component of depression, indicated by the up-regulation of pro-inflammatory cytokines. Pro-inflammatory cytokines are also well-known to regulate the enzymes of the kynurenine pathway (KP), which is responsible for metabolizing tryptophan, a precursor in serotonin synthesis. Enhanced pro-inflammatory cytokine levels may over-activate the KP, leading to tryptophan depletion and reduced serotonin levels, which can subsequently precipitate depressive symptoms. Therefore, such mechanism might represent a possible link between the endocannabinoid system (ECS) and the KP in depression, via the inflammatory and dysregulated serotonergic component of the disorder. This review will summarize the data regarding those natural and synthetic cannabinoids that increase pro-inflammatory cytokines. Furthermore, the data on such cytokines associated with KP activation will be further reviewed accordingly. The interaction of the ECS and the KP has been postulated and demonstrated in some studies previously. This review will further contribute to this yet less explored connection and propose the KP to be the missing link between cannabinoid-induced inflammation and depressive symptoms.

## 1. Introduction

Depression affects more than 264 million people around the world [1] and social, environmental, as well as genetic factors may contribute to its development. Substance abuse is among the most frequent causes in the development of depression [2]. Recently, cannabis and synthetic cannabinoid use has gained popularity, unfortunately their misuse as well at the same time [3,4,5]. Moreover, there is a great amount of clinical evidence demonstrating that such compounds can induce depressive symptoms [3,4,5,6,7,8,9,10,11].

As with many psychiatric disorders, in depression, multiple neurotransmitter pathways, endocrine systems, and brain regions are involved. The endocannabinoid system (ECS) and the kynurenine pathway (KP) have long been strongly implicated in this disorder. Both systems contribute to the neuroinflammatory and serotonin hypothesis of depression, which will be discussed later on. In fact, there is a growing body of evidence showing potential common points or clear evidence for the interaction of the ECS and the KP. These were discussed previously by our group [12,13,14] among others [15,16,17,18]. This review will further support the link between the ECS and KP in the aspect of depression by summarizing the data of pro-inflammatory cytokines, which can be regulated by exogenous cannabinoids and at the same time which can regulate the KP. Additionally, pro-inflammatory cytokines, the ECS and KP in general, and regarding their role in depression will be also briefly discussed. Reviewing such data will allow a better understanding of the effect of cannabinoids on the neuroinflammatory component of depression.

## 2. Background

### 2.1. Pro-Inflammatory Cytokines

Pro-inflammatory cytokines are small signaling proteins which up-regulate during inflammation, as they are crucial for initiating and promoting inflammatory responses to diseases [19,20]. They are predominantly produced by macrophages, but astrocytes, microglia, and neurons can also generate them in the brain [21]. Most notable pro-inflammatory cytokines are the interleukins (IL), like IL-1, 6, 8, 12, or 18, interferons (IFN), such as IFNγ and tumor necrosis factors (TNF), like TNFα [19]. They are able to freely pass through the blood–brain barrier (BBB), via multiple mechanisms such as passive diffusion through the leaky regions of the BBB, active transport, or via nerve fibers such as the vagus or trigeminal nerves [21]. They bind to cytokine receptors which can be divided into class I and class II based on their structural differences [22]. However, both classes uniformly activate the Janus kinase-signal transducers and activators of the transcription (JAK-STAT) pathway [22]. There is increasing evidence demonstrating that pro-inflammatory cytokines have a significant role in certain neurological and psychiatric disorders. For instance, in patients with schizophrenia, Alzheimer’s, or depression, pro-inflammatory cytokine levels are elevated or dysregulated [23]. In this review, depression will be discussed in this regard.

### 2.2. The Endocannabinoid System

The ECS has a crucial role in depression, confirmed by behavioral, anatomical, electrophysiological, and genetic evidence reviewed thoroughly elsewhere [24,25,26,27]. The ECS includes endogenous cannabinoids or endocannabinoids [28,29,30], the enzymes which synthesize and degrade endocannabinoids [31], and finally cannabinoid receptors, namely type 1 and 2 (CB_1_R and CB_2_R) which mediate the effects of endogenous or exogenous cannabinoids [32,33,34,35]. Both CBRs belong to the G-protein coupled receptor (GPCR) family generally coupling to G_αi_ type G-proteins. Accordingly, they inhibit adenylyl cyclase activity, leading to the presynaptic release inhibition of neurotransmitters such as γ-aminobutyric acid (GABA), dopamine, or acetylcholine [29,36,37,38]. On the other hand, there are data demonstrating other signaling pathways activated by CBRs, involving cell and G-protein type specificity or G-protein independent mechanisms [32].

The CB_1_R is the most abundant GPCR in the human brain, with higher expression levels compared to other GPCRs. Apart from the central nervous system (CNS), CB_1_Rs are found in several peripheral tissues, such as adipocytes, gastrointestinal tract, or the reproductive system [39]. Thus, CB_1_Rs are responsible for multiple physiological processes such as mood, appetite, food intake, thermoregulation, cognition, and memory [29,38,39,40,41]. CB_2_Rs are expressed mainly in cells of the immune system in the periphery [39], but they can be found in the CNS as well, for instance, in the brainstem or cerebellum [42]. CB_2_Rs have a significant role in the maintenance of homeostasis, analgesia, controlling cell proliferation, differentiation, and survival of neuronal and non-neuronal cells [39,43,44].

Cannabinoid receptors—apart from endocannabinoids—are also activated by natural (or plant-derived), semi, or fully synthetic exogenous cannabinoids. Among the plant-derived or phytocannabinoids, ∆^9^-tetrahydrocannabinol (THC), the major psychoactive component of cannabis, and the non-psychoactive cannabidiol (CBD) are the most studied and well-known. The most relevant structural classes of synthetic cannabinoids (SC) are aminoalkylindoles (e.g., WIN 55212-2), naphtholylindoles (e.g., JWH-018), and cyclohexylphenols (e.g., CP 55940) [45,46]. Together with the CBR antagonists/inverse agonist diaryl-pyrazole derivatives, such as rimonabant [47], SCs significantly contributed to the pharmacological mapping of the ECS. Since SCs greatly mimic the effects of cannabis, aminoalkylindoles, cyclohexylphenols, and naphtholylindoles especially, are the most common SCs found in the K2/Spice products, which are the most widely abused class of drugs nowadays [11,48,49]. Indole, indazole carboxamides structured SCs (e.g., AB-PICA and AB-PINACA, respectively) were joined to this class very recently [46].

As mentioned previously, there are numerous preclinical and clinical studies pointing out that cannabis/THC and SCs use significantly contributes to the development of depression [3,6,8,50,51,52,53]. In fact, a recent study showed that SC users displayed a higher Beck Depression Inventory score [54] compared to natural cannabis users, with similar socio-demographic characteristics [51]. Another study pointed out a similar outcome, where SC use was associated with increased mental health symptomatology—including depression—compared to natural cannabinoid use [10]. These findings can be explained by the higher CB_1_R affinity and agonist potency of the SCs compared to THC [52,55].

### 2.3. The Kynurenine Pathway

**Figure 1:**
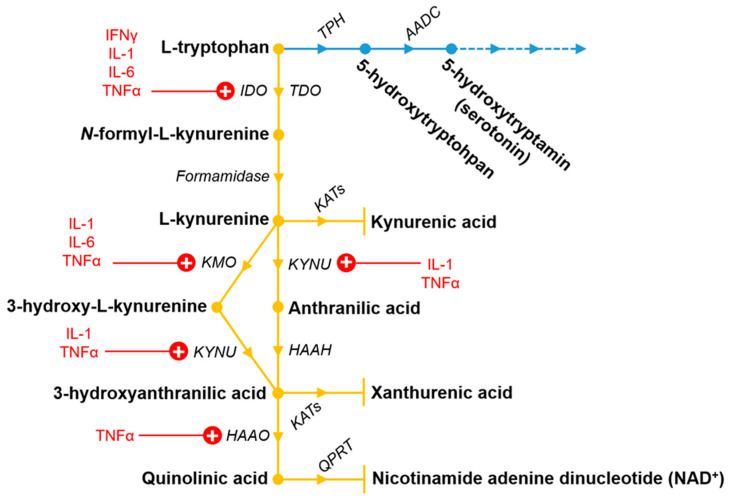
The tryptophan metabolic pathway including the kynurenine (yellow) and partly the serotonin pathway (blue). Pro-inflammatory cytokines discussed in this review which upregulate (highlighted by “+”) the different enzymes are also indicated in red. For further information, see Section 4 and Table 2. Metabolites and enzymes of the pathways are shown in bold and italic, respectively. The dashed lines in the serotonin pathway indicates the further continuation of the pathway, which is not discussed here. Abbreviations: AADC: aromatic acid decarboxylase enzymes; HAAH: 3-hydroxyanthranilic acid hydroxylase; HAAO: 3-hydroxyanthranillic acid 3,4-dioxygenase; IDO: indoleamine 2,3-dioxygenase; KATs: kynurenine aminotransferases; KMO: kynurenine 3-monooxygenase; KYNU: kynureninase; QPRT: quinolinic acid phosphoribosyltransferase; TDO: tryptophan 2,3-dioxygenase; TPH: tryptophan hydroxylase.

Tryptophan (Trp) is an essential amino acid, which is pivotal in the brain and in mammalian cells, and is mainly metabolized via the KP (Figure 1). Kynurenic acid (KYNA) is one of the most studied and clinically relevant metabolite of the KP. KYNA is an endogenous glutamate receptor antagonist, which has neuroprotective effects and is produced by kynurenine aminotransferases (KAT)s from l-kynurenine (L-KYN) mostly in astrocytes [56,57,58]. L-KYN is formed by formamidase enzyme from *N*-formyl-l-kynurenine, which is created from l-Trp by two enzymes, namely the tryptophan 2,3-dioxygenase (TDO) and the indoleamine 2,3-dioxygenase 1 and 2 (IDO1 and IDO2). It is well known, that l-KYN can transform not just to KYNA, but is also able to convert into anthranilic acid by kynureninase and to 3-hydroxykynurenine by kynurenine 3-monooxygenase (KMO). Anthranilic acid can be further converted to 3-hydroxyanthranilic acid by 3-hydroxyanthranilic acid hydroxylase. In addition to this, 3-hydroxykynurenine can also convert to 3-hydroxyanthranilic acid by kynureninase enzyme. Besides that, 3-hydroxykynurenine can modify to xanthurenic acid, as well. Additionally, 3-hydroxyanthranillic acid further transforms to quinolinic acid (QUIN) by 3-hydroxyanthranillic acid 3,4-dioxygenase. In the end of the KP, QUIN is degraded to nicotinamide adenine dinucleotide by quinolinic acid phosphoribosyltransferase. Opposite to KYNA, QUIN is an endogenous glutamate receptor agonist produced by microglia [59] and it can cause lipid peroxidation [60] and has a relevant role in the neurodegenerative process [61,62].

### 2.4. The Serotonin and Inflammatory Hypothesis of Depression: Possible Links between ECS and KP in Depression?

The serotonin hypothesis was introduced more than 50 years ago as a possible pathological background mechanism for depression [63]. The hypothesis refers to a dysregulated serotoninergic system, implicating reduced levels of serotonin, serotonin transporters and/or receptors in patients with depression [64,65,66,67,68,69,70]. It has been long since described that reduced serotonin levels are due to Trp depletion [71]. Serotonin or 5-hydroxytryptamine is metabolized from Trp through 5-hydroxytryptophan catabolized by the tryptophan hydroxylase and aromatic acid decarboxylase enzymes [71] (Figure 1). However, only a small fraction of the Trp pool is converted to serotonin, the vast majority (~95%) is metabolized via KP, as discussed in the previous section. Thus, even a small change in the activity of the KP can have a significant impact on the Trp pool in the brain [72,73]. Indeed, there is numerous clinical evidence showing that there is an imbalance in the metabolism of KP in depression. The amount of Trp, l-KYN, and KYNA, for instance, is decreased in the serum and plasma of patient with depression, whereas QUIN is increased. These data have been reviewed in detail previously [74,75,76,77,78,79,80]. There are also several genetic mutations in the KP, which are connected to depression. Some polymorphisms of the IDO1, 2 and KMO encoded genes are identified in patients with depression [81]. On the other hand, data on increased levels of KP metabolites in blood serum and CSF in individuals with depressive disorders have been inconsistent [82,83,84,85]. It has been also proposed that changes in enzymes and metabolites of the KP are not necessarily parallel to events in the brain [86]. These data also clearly show the complexity of depression and that the serotonin hypothesis is not the only background mechanism responsible for this psychiatric disorder.

**Figure 2:**
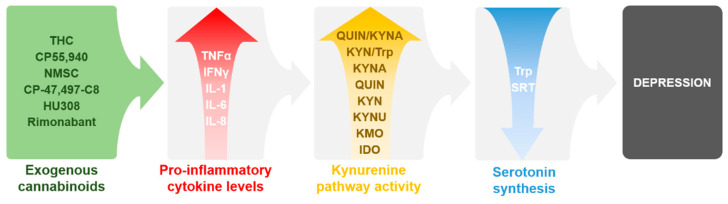
Summary of the reviewed data and their proposed connections between exogenous cannabinoids, pro-inflammatory cytokines, KP, and depression as discussed in the sections below and in Table 1 and Table 2. Abbreviations: IDO: indolamine 2,3-dioxygenase; IFNγ: interferon γ; IL: interleukin; KMO: kynurenine 3-monooxygenase, KYN: l-kynurenine, KYNA: kynurenic acid; QUIN: quinolinic acid; THC: ∆^9^-tetrahydrocannabinol; TNFα: tumor necrosis factor α; Trp: tryptophan; SRT: serotonin.

There are multiple studies showing that depression also consists of an inflammatory component centering not just in the brain but throughout the body. One of the main indications of such mechanism is the significant enhancement of circulating pro-inflammatory cytokines in animal models of depression and also in patients with depression, which can be reversed by antidepressants. These findings have been previously reviewed extensively [21,87,88,89,90,91,92]. It is well known that enzymes of the KP, especially IDO, can be activated by pro-inflammatory cytokines, which may lead to Trp depletion and possibly depression as described above. More interestingly, there are numerous data demonstrating that exogenous cannabinoids can enhance the levels of pro-inflammatory cytokines, which may over activate the KP, potentially leading to depression. These findings will be discussed later on. Therefore, there is a potential link between cannabinoids and the KP in depression, where exogenous cannabinoids potentially induce inflammation by increasing pro-inflammatory cytokines. Such effect then enhances the activity of the KP leading to Trp depletion and reduced levels of serotonin, which eventually may contribute to depression (Figure 2). This link might be a possible explanation for depressive episodes induced by natural and synthetic cannabinoids misuse. The following sections will discuss those exogenous cannabinoids which are known to increase pro-inflammatory cytokines. Additionally, such cytokines which have been associated with the activation of the KP in neuroinflammation and/or depression will be further reviewed accordingly.

## 3. Cannabinoids That Enhance Pro-Inflammatory Cytokine Levels

Cannabinoids, endogenous, synthetic, and natural types have been generally associated with anti-neuroinflammation by downregulating pro-inflammatory and/or upregulating anti-inflammatory cytokines typically through CB_2_Rs [93,94,95]. However, there is growing evidence demonstrating that natural and synthetic cannabinoids can indeed upregulate pro-inflammatory cytokines and thus possibly induce neuroinflammation and/or depression. This section will review these data (see Table 1). There is also substantial evidence that cytokines can induce mood alterations by regulating cannabinoid receptors [96,97,98,99], however this is out of the scope of this current review.

### 3.1. Natural Cannabinoids (Cannabis, THC, and CBD)

THC and CBD have been long known to regulate cytokine levels in a concentration dependent manner. In an earlier study in human peripheral blood mononuclear cells, THC and CBD in concentrations comparable to plasma levels prior to smoking marijuana (10–100 ng/mL), increased the concentration of IFNγ, while in higher concentrations (5–20 μg/mL), fully blocked the synthesis and/or release of this cytokine [100]. Another study also pointed out the biphasic effect of THC on cytokine regulation in mononuclear cells: TNF-α and IL-6 synthesis was inhibited by 3 nM THC but stimulated by 3 μM, as was with IFNγ synthesis [101]. Other studies also showed that the biphasic effect of THC on pro-inflammatory cytokines seems to be not only dependent on concentration, but also whether the experimental animals are naïve or neuroinflammation was induced [94,95,102,103,104,105]. In eosinophilic leukemia cell lines, both THC and CBD significantly increased IL-8 production, while in human T-lymphotropic virus type 1 (HTVL-1) positive B cell lines, only THC increased IL-8 levels [106]. Cutando and co-workers showed that subchronic administration of THC to mice activated cerebellar microglia and increased the expression of IL-1β and TNFα genes [107]. The neuroinflammation induced by THC was reversed by inhibiting IL-1β receptor signaling [107]. It is worth noting that CBD has a peculiar pharmacological profile which differs from THC and other natural and synthetic cannabinoids. In vitro and in vivo studies have indicated that CBD may act as a negative allosteric modulator of CB_1_R and an agonist of CB_2_R, transient receptor potential vanilloid 1 (TRPV1), 5-hydroxytryptophan_1A_ receptors, and peroxisome proliferator-activated receptors γ (PPARy) [108]. Such multi-targeted action can help explain a prevailing anti-inflammatory action of CBD in vivo and in vitro, as reviewed elsewhere [109,110]. Briefly, CBD reduces stress and LPS-stimulated release of pro-inflammatory cytokines [109]. This anti-inflammatory effect could counteract THC-induced inflammation, thus explaining the beneficial profile of CBD in attenuating some detrimental effects of THC and in treatment conditions associated with drug abuse and dependence [111,112].

It is widely accepted that adolescence is a vulnerable period in terms of THC exposure, which can later result in psychiatric disorders in adulthood [50]. Additionally, multiple studies associated this with neuroinflammation, in particular with regulating cytokine expression. Moretti and co-workers showed that IL-1β and TNFα gene and protein expression increased in peripheral macrophages following chronic THC exposure in adolescent mice. Such was not the case when adult mice were treated chronically [113]. In fact, the opposite was observed if the same cytokines were analyzed right after the final THC treatment in both adolescent and adult animals [113]. Later on, the same findings were also confirmed in the hippocampus and hypothalamus by the same group [114]. Another study investigated the chronic effect of THC consumption in adolescent female rats. Here, the THC treatment enhanced expression levels of TNFα in microglia of the prefrontal cortex which was associated with depression-like phenotype [115].

Cannabis use disorder has its own set of definitions for diagnosis as it has been included in the latest edition of Diagnostic and Statistical Manual of Mental Disorders (DSM-5) [116]. It has been recently demonstrated that patients with cannabis use disorder have increased serum levels of IL-1β, IL-6, IL-8, and TNF-α levels [117]. In another study, they compared physically active chronic cannabis users (at least once per week for the past 6 months) and non-users in terms of the presence of depression and immune health indicated partly by IL-6 [118]. However, they found no difference between the two groups in IL-6 serum levels.

### 3.2. Semi- and Fully Synthetic Cannabinoids

Data regarding synthetic cannabinoids and cytokine regulation are relatively recent, but limited. CP55940 is functionally and structurally analogue to JWH-018 and to CP47497 which is a frequent component of “K2/Spice” synthetic cannabinoid blends [119]. In a study involving promyelocytic cells HL-60 transfected with CB_2_R, CP55940 increased TNFα mRNA after 1 h and protein levels after 24 h [120]. Both effects were CB_2_R mediated [120]. Very recently, Zawatsky and co-workers have shown that oropharyngeal administration of the synthetic cannabinoid CP55940 to mice significantly increased the mRNA levels of CB_1_Rs and induced the expression levels of IL-1β, IL-6, and TNFα in the lung [119]. In another study, they investigated a representative member of cyclohexylphenols of SCs which can bind to both CBRs, namely CP-47497-C8 (cannabicyclohexanol). Cannabicyclohexanol was also found in “Spice” in Germany and Japan [4,121] and was described to increase IL-6 and TNFα levels in peripheral blood mononuclear cells [122]. A CB_2_R selective synthetic cannabinoid agonist, HU308 in human primary leukocytes, was shown to induce the secretion of IL-6 via G_αS_ coupled signaling [43]. The semi-synthetic CBD derivative 2-(methylsulfonamido)ethyl cannabidiolate (NMSC) enhanced IL-1β and IL-6 mRNA levels in RAW264.7 macrophages upon IL-17 stimulation, but only in higher concentration (10 µmol/L) [123]. In lower concentration (5 µmol/L), it showed the opposite effect.

An interesting study was conducted with the CB_1_R selective antagonist/inverse agonist rimonabant, which was withdrawn from the market due to its adverse psychiatric side-effects, including depression, anxiety, and suicidal ideation after long-term usage [124]. Such clinical data were strengthened by pre-clinical in vitro results. Namely, in rats which showed depressive-like phenotype, long-term rimonabant treatment increased the level of IL-6 and TNFα in the medial prefrontal cortex and in the hippocampus, respectively [125]. In a very recent study, they investigated the level of inflammation apart from oxidative stress and DNA damage in 40 synthetic cannabinoid (the exact compounds were not determined) addicts and they found that IL-1β, IL-6, and TNF-α serum cytokine levels were significantly higher compared to the healthy groups [126].

**Table 1:** Summary of cannabinoids known to increase pro-inflammatory cytokines.

Cannabinoid	Cytokine	Studied Sample	Ref.
THC	IFNγ	PMBC	[100,101]
	TNFα	PMBC	[101]
		Adult mouse peripheral macrophage	[113]
		Adult mouse hippocampus and hypothalamus	[114]
		Female adol. rat microglia PFC	[115]
	IL-1	Microglia	[107]
		Adult mouse peripheral macrophage	[113]
		Adult mouse hippocampus and hypothalamus	[114]
	IL-6	PMBC	[101]
	IL-8	Eosinophilic leukemia cell line and HTLV-1 positive B cell line	[106]
CBD	IFNγ	PMBC	[101]
	IL-8	Eosinophilic leukemia cell line	[106]
Cannabis	IL-1, IL-6, IL-8	Serum from patients with CUD	[117]
CP55940	TNFα	HL-60 transfected with CB_2_R; mouse lung	[119,120]
	IL-1	Mouse lung	[119]
	IL-6	Mouse lung	[119]
NMSC	IL-1β, IL-6	RAW264.7 macrophage	[123]
CP-47497-C8	TNFα, IL-6	PMBC	[122]
HU308	IL-6	Human primary leukocytes	[43]
Rimonabant	TNFα	rat hippocampus	[125]
	IL-6	rat mPFC	[125]

CUD: cannabis used disorder; HTVL-1: human T-lymphotropic virus type 1; PFC: prefrontal cortex; PMBC: peripheral mononuclear cells; mPFC: medial prefrontal cortex; NMSC: 2-(methylsulfonamido)ethyl cannabidiolate.

## 4. Pro-Inflammatory Cytokines Parallelly Up-Regulated with the KP in Neuroinflammation and/or Depression

As we saw in the previous section, there are multiple studies pointing out the upregulation of IFNγ, IL-1, IL-6, IL-8, and TNFα pro-inflammatory cytokines via cannabinoid induction. This section will review the data regarding the effect of the above- mentioned cytokines on the regulation of the KP enzymes and their metabolite production. There are other reviews describing the relationship between cytokines and the KP in different disorders [127,128,129,130]. However, this section is the first to thoroughly review these data in the aspect of neuroinflammation or depression. Table 2 summarizes the data discussed below. Important to note that in contrast to multiple reports, a previous study showed reduced KP metabolism and pro-inflammatory cytokine levels in post mortem ventrolateral prefrontal cortex tissues from individuals with depressive illness [131]. The study also discussed that such unexpected result might be due to the different specific brain region investigated and/or the distinct diagnosis classification of the depressed samples used, which might have influenced the overall results. Finally, the study also pointed out that the regulation of KP in the human brain might be brain-region specific in depression.

### 4.1. IFN-γ

IFN-γ has long been known for regulating IDO activity [132,133], which has been discussed extensively in a previous review in 2014 [127]. Since then, additional research has been done in this area. The synergistic effect between IFN-γ and IL-1 is well-known in regulating IDO enzyme activity and transcription [134,135,136]. Moreover, in THP-1 human monocytic cell lines, Fujigaki and co-workers also demonstrated that LPS-induced IDO enzyme activity was upregulated when IFN-γ together with IL-1β, IL-6, and TNFα were present [137]. However, galectins, which also play an important role in neuroinflammation, and corticosteroids have also been shown to enhance the effect of IFN-γ in controlling IDO expression. In the mouse hippocampus, it has been shown that galectin-9, dexamethasone, corticosterone, and aldosterone interacted with IFN-γ to further enhance the mRNA expression of different IDO variants [138,139]. In a chronic social defeat mouse model, which models the anhedonic and social-avoidance aspect of depression, IFN-γ plasma levels increased together with KYN, 3-HK [140]. On the other hand, KYNA plasma levels were also enhanced, which seem to be in contrast with the elevated QUIN/KYNA ratio attributed to depression. The study did not further elaborate on this result. Another animal model of depression, the chronic mild stress procedure significantly increased IFNγ and IDO mRNA and decreased KAT II mRNA in the rat cortex [141]. The latter case may project ahead the increased QUIN/KYNA ratio observed in depression, since due to reduced KAT II availability, KYN conversion is more likely to be directed towards QUIN rather than KYNA production.

### 4.2. IL-1

Apart from IFN-γ, IL-1 is the most significant pro-inflammatory cytokine to regulate IDO. IL-1 alone transcriptionally activates the IDO gene in primary macrophages and is able to enhance the activity of the enzyme but only in the IFN-γ pretreated THP1 monocytic leukemia cell line [142].

Fractalkine receptor (CX_3_CR1) deficient mice have been demonstrated to display depressive-like behavior following LPS treatment [143]. In such mice, increased microglial mRNA expression of IL-1β, IDO, and KMO after LPS treatment was observed [143]. In a Bacille Calmette Guérin (BCG) depressive-like behavior mouse model, both IL-1β and KMO but not IDO-1 and -2 mRNA were upregulated in microglia [144]. Upon LPS stimulation, mRNA expression of IL-1β was dose-dependently increased parallelly with IDO-1 and KMO in murine microglia [145]. Additionally, KMO deletion prevented the LPS-induction of IL-1β.

Laumet and co-workers demonstrated the involvement of IL-1β in nerve injury-induced depression associated with enhanced KMO mRNA brain expression and activity in mice brain [146]. Additionally, functional IDO-positive dendritic cells produced significantly more IL-1β than IDO-negative cells upon CD40L stimulation [147]. IL-1β treatment in human hippocampal progenitor cells induced the transcription of IDO, KMO, and KYNU, which resulted in an increase in KYN production [148]. In the same study, inhibiting the KMO enzyme reversed the reduction of neurogenesis in human hippocampal progenitor cells induced by IL-1β. In another study involving the hippocampus, IDO1 mRNA expression was also enhanced by the upregulation of IL-1β production in the hippocampus of rats with coexisting chronic temporal lobe epilepsy and depressive behavior [149]. The two forms of the alarmin protein, high mobility group box-1 (HMGB1)—the fully reduced (fr-HMGB1) and the disulfide (ds-HMGB1) form—are known to induce depressive-like behavior [150]. Recently, it has been shown in mouse hippocampal tissues ds-HMGB1 directly activated IDO, KMO, and KYNU in parallel with IL-1β upregulation [151]. With fr-HMGB1, the same observations were made following H_2_O_2_ treatment. In the study, both forms of HMGB1 induced depressive-like behavior.

### 4.3. IL-6

The correlation between IL-6 and KP metabolites and enzymes has been long known. For instance, lower Trp levels in patients with depression are known to be inversely correlated to serum concentrations of IL-6 [152]. There is evidence that depressive and anxiety symptoms in the early puerperium in fare causally related to an increased catabolism of Trp into KYN, which may be associated with increased plasma levels of IL-6 [153]. Schwieler showed that in patients with unipolar treatment-resistant depression, IL-6 plasma levels and the QUIN/KYNA ratio in the plasma significantly increased compared to healthy volunteers [154]. Kruse and co-workers demonstrated that in a human experimental model of endotoxin-induced depressed mood, there was a positive correlation in plasma concentrations of KYN and QUIN and IL-6. However, changes in the KP metabolites did not mediate the correlation between cytokines and depressed mood [80]. In a recent study, IL-6 and QUIN plasma levels were positively correlated in women with peripartum onset depression (PPD) [155]. In another recent study with frail patients, it was found that the KYN/Trp ratio and KYN levels were strongly correlated with IL-6 plasma levels [156]. The authors concluded that these results are in accordance with the serotonin-KYN hypothesis of depression and also may explain the high prevalence of depression among individuals with frailty status [156].

IL-6 may also contribute to cortisol’s induction of TDO, as increased IL-6 in depression are implicated in elevated hypothalamic–pituitary–adrenal activity and cortisol levels, which in turn activates TDO [157,158]. Bay-Richter and colleagues found that cerebrospinal fluid levels of QUIN and KYNA increased and decreased, respectively, in suicide attempters, which remained over time and also high IL-6 cerebrospinal fluid levels correlated with more severe suicidal symptoms [159].

In another study with microglia, LPS stimulation dose-dependently increased the mRNA expression of IL-6 and parallelly of IDO-1 and KMO [145]. There is also multiple evidence for IL-6 regulating KP enzymes in the brain. Kim and co-workers have shown that intra-hippocampal administration of IL-6 in rats induces IDO1 expression through the JAK/STAT pathway [160]. Xie and co-workers demonstrated in rats with coexisting chronic temporal lobe epilepsy and depressive behavior that the upregulation of IL-6 production in the hippocampus enhanced IDO1 mRNA expression too in the same brain area [149]. In rats with ovariectomy-induced depressive-like behavior, showed parallelly elevated IL-6 and IDO protein levels in the hippocampus [161]. In another model, the enhancement of LPS induced IDO and KMO mRNA expression was accompanied by a significant increase in IL-6 expression in the rat hippocampus and cortex and in cultured glial cells [162].

In the Netherlands Study of Depression and Anxiety (NESDA), a cohort consisting almost 3000 participants, no indications were found in KYN/Trp ratio for mediating the relationship between changes in IL-6 levels and depressive symptoms [83].

### 4.4. IL-8

Maes and colleagues demonstrated that hepatitis C patients who received IFNα treatment showed an increase in depressive symptoms and KYN/Trp quotient along with elevations in IL-8 plasma levels [163]. In the previously mentioned study, where they investigated the relation between KYNUs, immune activity and depressive and anxiety symptoms in the early puerperium, they observed enhanced IL-8 plasma levels parallelly with increased KYN/Trp quotient [153].

### 4.5. TNFα

TNFα and IDO serum levels parallelly increased in major depressive disorder (MDD) patients, which was reduced by post-treatment [164]. Chronic social defeat depression mouse model led to increased plasma levels of TNF-α in parallel with KYN, 3-HK, and KYNA as seen with IFNγ [140]. Similar to IL-6, there was also a positive correlation in plasma concentrations of KYN and QUIN and TNFα in the human experimental model of endotoxin-induced depressed mood. However, changes in the KP metabolites did not mediate the correlation between cytokines and the depressed mood [80]. TNFα levels correlated positively with QUIN plasma levels in women with PPD [155]. Haroon and co-workers found a correlation among peripheral and central KP metabolites and inflammation in depression in a study involving 72 unmedicated depressed patients. Accordingly, plasma TNFα was robustly associated with plasma KYN and KYN/Trp ratio levels, which was in turn significantly correlated with CSF KYN, KYNA, and QUIN [165]. Additionally high TNFα-kynurenine/tryptophan subjects showed enhanced depression severity, anhedonia, and treatment nonresponse [165]. In the study involving frailty patients, they also found that the KYN/Trp ratio and KYN levels were strongly correlated with TNFα and TNFαR1 levels too, while Trp and KYNA alone were also strongly correlated with TNFαR1 levels [156].

O’Connor and co-workers demonstrated that in the BCG mice model, TNFα, IDO, and HAO mRNA significantly increased in the brain [166]. The same group also showed that IFNγ and TNFα synergistically induce IDO in primary microglia cells and they are both necessary for the induction of IDO and depressive-like behavior in mice after BCG infection [167]. There is also further evidence that TNFα together with IFNγ can transcriptionally activate IDO [136,168,169].

The enhancement of LPS induced IDO and KMO expression was also associated with increased TNFα expression in the rat hippocampus and cortex and in cultured glial cells [162]. In murine microglia upon LPS stimulation, mRNA expression of TNF-α was dose-dependently increased together with IDO1 and KMO mRNA, and KMO deletion eliminated the LPS-induced TNFα elevation [145]. The two forms of the high mobility group box-1 (HMGB1) protein (fr-HMGB1 and ds-HMGB1) mentioned regarding IL-1β cytokine, are also known to upregulate TNFα besides inducing depressive-like behavior [150]. Additionally, alongside IL-β, TNFα was also upregulated in mice hippocampal tissues in parallel with IDO, KMO, and KYNU, which were activated by both forms of HMGB1 [151].

**Table 2:** Summarizing the data regarding upregulated pro-inflammatory cytokines associated with altered KP enzymes or metabolites.

Cytokine	KP Enzyme or Metabolite	Studied Sample	Comment	Ref.
IFNγ	IDO mRNA ↑	mouse hippocampus	galectin-9 synergism	[138]
		mouse hippocampus	dexamethasone, corticosterone and aldosterone synergism	[139]
		rat cortex	CMS model	[141]
	KAT II mRNA ↓	rat cortex	CMS model	[141]
	KYN, 3-HK, KYNA ↑	mouse plasma	CSD model	[140]
IL-1	IDO mRNA ↑	primary macrophage		[142]
	IDO activity ↑	THP1 monocytic leukemia cell line	IFNγ pretreatment	[142]
	IDO, KMO mRNA ↑	CX3CR1 K.O. mouse microglia		[143]
	KMO mRNA ↑	mouse microglia	BCG model	[144]
	IDO, KMO mRNA ↑	murine microglia	LPS-induction	[145]
	KMO mRNA ↑	mouse brain	nerve injury-induced depression	[146]
	IDO, KMO, KYNU mRNA, KYN ↑	human hippocampal progenitor cells		[148]
	IDO1 mRNA ↑	rat hippocampus	coexisting chronic temporal lobe epilepsy and depressive behavior	[149]
	IDO, KMO and KYNU activity ↑	mouse hippocampus	HMGB1 induced depressive like behavior model	[151]
IL-6	Trp ↓	human serum	in patients with depression	[152]
	KYN ↑	female human serum	in early puerperium associated with anxiety and depression	[153]
	QUIN/KYNA ratio ↑	human plasma	in patients with unipolar treatment-resistant depression	[154]
	KYN and QUIN ↑	human plasma	did not mediate the correlation between cytokines and depressed mood	[80]
	QUIN ↑	female human plasma	women with PPD	[155]
	KYN/Trp ratio, KYN ↑	plasma from frailty patients	may explain high prevalence depression in frailty patients	[156]
	QUIN ↑ KYNA ↓	human CSF	in suicide attempters	[159]
	IDO1, KMO mRNA ↑	murine microglia	following LPS-stimulation	[145]
	IDO1 protein ↑	rat hippocampus	through JAK/STAT pathway	[160]
	IDO1 mRNA ↑	rat hippocampus	coexisting chronic temporal lobe epilepsy and depressive behavior	[149]
	IDO1 protein ↑	rat hippocampus	ovariectomy-induced depression model	[161]
	IDO, KMO mRNA ↑	rat hpc., ctx., and cultured glia cells		[162]
IL-8	KYN/Trp quotient ↑	human plasma	IFNα-induced depressive symptoms	[163]
		female human serum	in early puerperium associated with anxiety and depression	[153]
TNFα	IDO ↑	human serum	in MDD patients	[164]
	KYN, 3-HK, KYNA ↑	mouse plasma	CSD mouse model	[140]
	KYN and QUIN ↑	human plasma	did not mediate the correlation between cytokines and depressed mood	[80]
	QUIN ↑	female human plasma	women with PPD	[155]
	KYN, KYN/Trp ratio ↑	plasma	associated with enhanced depression, anhedonia, and treatment nonresponse	[165]
	KYN, KYNA, QUIN ↑	CSF	in unmedicated depressed patients	[165]
	KYN/Trp ratio, KYN ↑	plasma from frailty patients	may explain high prevalence depression in frailty patients	[156]
	IDO, HAAO mRNA ↑	mouse brain	BCG model	[166]
	IDO activity ↑	mouse microglia cells	BCG model	[167]
	IDO, KMO mRNA ↑	rat hpc., ctx., and cultured glia cells		[162]
	IDO1, KMO mRNA ↑	murine microglia	following LPS-stimulation	[145]
	IDO, KMO and KYNU activity ↑	mouse hippocampus	HMGB1 induced depressive like behavior model	[151]

↑: increase; ↓: decrease; BCG: Bacille Calmette Guérin mice model of depression; CMS: chronic mild stress model; ctx.: cortex; CSD: chronic social defeat model; HMGB1: high mobility group box-1 protein; hpc.: hippocampus; MDD: major depressive disorder; PPD: peripartum onset depression.

## 5. Summary and Conclusions

This paper summarized pre-clinical and clinical evidence on pro-inflammatory cytokines which are upregulated by natural and synthetic cannabinoids, thus might be contributing to the inflammatory component of depression induced by such compounds. Additionally, the manuscript further reviewed those cytokines which are parallelly upregulated with certain enzymes and metabolites of the KP, possibly leading to the over-activation of the KP. This over-activation may significantly contribute to the downregulated serotoninergic system attributed to depression.

Cannabis use has been increasing rapidly over the past few years, due to its legalization in a growing number of US states and other countries. SC consumption has also gained significant popularity over the years, however, in contrast to natural cannabinoids, for many SCs, the receptor preference, affinity, metabolic mechanisms, and pharmacodynamics are unknown [45,46]. Additionally, the discrepancies in the pro- and anti-inflammatory effects of THC and CBD are known and it is explained by the difference in the applied concentrations and model systems (naïve vs. inflammatory-induced) and by the complex pharmacological profile in case of CBD. The investigation on the molecular mechanisms by which cannabinoids could lead to increased inflammatory effects could potentially unravel important targets for controlling neuroinflammation associated with drug abuse and dependence and its emotional consequences. Both natural and synthetic cannabinoids significantly contribute to the development of depression based on multiple pre-clinical and clinical studies. The data reviewed here may reveal a possible link between the ECS and the KP and help to overview the connection between cannabinoids, inflammation, and KP in relation to the pathophysiology of depression. Although, there is no direct evidence so far that exogenous cannabinoids induce depression via inflammation-stimulated KP in one experimental system, the data gathered in this review clearly demonstrate its strong possibility. Nevertheless, reviewing such data may raise interest to study the inflammatory component of depression by pharmacological and/or genetic manipulation of either the ECS or the KP. Selective exogenous cannabinoids [170,171] and enzyme inhibitors of the KP as well as CBR and KP enzyme knock-out animals are available and widely used to study the function of the ECS and KP [172,173,174,175,176,177,178,179]. Applying these tools may reveal the response of each system to one another when manipulated in inflammatory-induced depression.

## Data Availability

Not applicable.
